# Predicting the cumulative risk of death during hospitalization by modeling weekend, weekday and diurnal mortality risks

**DOI:** 10.1186/1472-6963-14-226

**Published:** 2014-05-21

**Authors:** Enrico Coiera, Ying Wang, Farah Magrabi, Oscar Perez Concha, Blanca Gallego, William Runciman

**Affiliations:** 1 Centre for Health Informatics, Australian Institute for Health Innovation, University of New South Wales, Sydney 2052, Australia; 2 The School of Psychology, Social Work & Social Policy, University of South Australia, Adelaide, Australia; 3 Australian Patient Safety Foundation, Adelaide, South Australia, Australia

**Keywords:** Weekend effect, Risk exposure model, Dynamic risk prediction, Quality of hospital service

## Abstract

**Background:**

Current prognostic models factor in patient and disease specific variables but do not consider cumulative risks of hospitalization over time. We developed risk models of the likelihood of death associated with cumulative exposure to hospitalization, based on time-varying risks of hospitalization over any given day, as well as day of the week. Model performance was evaluated alone, and in combination with simple disease-specific models.

**Method:**

Patients admitted between 2000 and 2006 from 501 public and private hospitals in NSW, Australia were used for training and 2007 data for evaluation. The impact of hospital care delivered over different days of the week and or times of the day was modeled by separating hospitalization risk into 21 separate time periods (morning, day, night across the days of the week). Three models were developed to predict death up to 7-days post-discharge: 1/a simple *background risk* model using age, gender; 2/a *time-varying risk* model for exposure to hospitalization (admission time, days in hospital); 3/*disease specific* models (Charlson co-morbidity index, DRG). Combining these three generated a full model. Models were evaluated by accuracy, AUC, Akaike and Bayesian information criteria.

**Results:**

There was a clear diurnal rhythm to hospital mortality in the data set, peaking in the evening, as well as the well-known ‘weekend-effect’ where mortality peaks with weekend admissions. Individual models had modest performance on the test data set (AUC 0.71, 0.79 and 0.79 respectively). The combined model which included time-varying risk however yielded an average AUC of 0.92. This model performed best for stays up to 7-days (93% of admissions), peaking at days 3 to 5 (AUC 0.94).

**Conclusions:**

Risks of hospitalization vary not just with the day of the week but also time of the day, and can be used to make predictions about the cumulative risk of death associated with an individual’s hospitalization. Combining disease specific models with such time varying- estimates appears to result in robust predictive performance. Such risk exposure models should find utility both in enhancing standard prognostic models as well as estimating the risk of continuation of hospitalization.

## Background

Prognostic models help clinicians identify those patients most at risk of negative outcomes, and tailor therapy to manage that risk [1,2]. Such models are typically developed for specific conditions or diseases, combining clinical indicators (such as patient or disease characteristics) and test results that stratify populations by risk [3]. Performance varies and even the best models often have good but not high classification accuracy [2]. Models are most often developed using data from specific periods of time, patient cohorts or geographies, and when they are evaluated against new populations, may not perform as well as in the original population [3].

Variation in predictive accuracy of models in part must be due to variations in disease patterns across populations, but is also likely due to local variations in clinical service and practice. If prognostic models specifically incorporated information about health service delivery the result might be more accurate, generalizable clinical tools. There are for example well known risks associated with hospitalization that can lead to patient harm or death [4]. These risks vary with diagnosis [5,6], hospital [7,8] and route of admission [9]. Risks also are known to vary significantly with the time that hospitalization occurs, with weekend admissions carrying greater risk than other days [10-17].

Modeling the risk of exposure to hospitalization should allow for more informed decisions around the decision to admit or discharge. Indeed, whilst it is standard to develop risk-benefit models for new interventions such as medicines or procedures, we do not routinely apply the same logic to the decision to admit to or discharge from hospital, one of the most universal of all clinical interventions. In the same way that radiation exposure models help determine when a patient has exceeded a safe radiation dose, it should be feasible to develop models that determine when patients have exceeded a safe ‘dose’ of hospitalization.

Some work has explored the risk of harm to a patient following exposure to an average day in hospitalization [10,18]. Combining models which predict time-varying risks associated with hospitalization with traditional disease-specific prediction rules should theoretically result in much more accurate predictive tools which can be used to update risk estimates as an admission progresses over time. In this paper we report on the development and evaluation of one such family of models, using only standard administrative data.

## Methods

### Data

All admissions to 501 public and private hospitals in New South Wales (NSW) Australia between 1 July 2000 and 30th June 2007 were extracted from the NSW Admitted Patient Data Collection (APDC). Admissions were coded using the International Classification of Diseases 10th revision Australian modification (ICD-10-AM) and Australian refined diagnosis related groups (DRG) [19]. Records with an invalid or missing admission date, date of death, principal diagnosis, DRG, patient age or gender were excluded. After exclusion, a total of 11,732,260 admissions remained, with 201,647 deaths either during hospitalization (177,828) or within 7 days of discharge (23,819). A Charlson comorbidity index was calculated for each admission using ICD-10-AM codes [20].

Hospital mortality rates are prone to “discharge bias”, underestimating the true impact of hospitalization on death rates because some deaths occur post-discharge [21]. To capture such post-discharge deaths, admission records were linked to the death registry [22,23].

**Figure 1:**
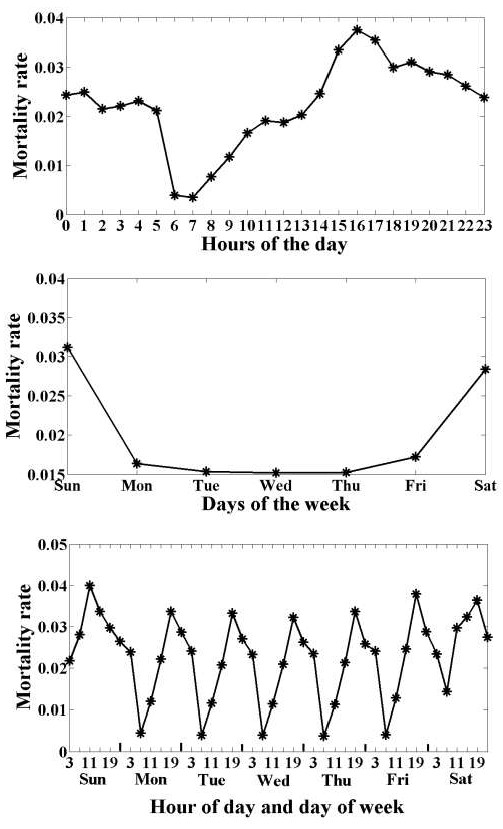
The probability of death up to seven days post-admission varied by the day of admission and by the time of the day of admission.

Inspection of the data set revealed that the probability of death up to seven days post discharge varied by day of admission, with weekend admissions having a higher risk of mortality [13-17]. The data also showed that the risk of mortality varied by the time of day of admission, peaking in the early evening and lowest in the morning. Combining these two revealed a fine-grained sinusoidal pattern to the risk of mortality with both daily and weekly periodicities (Figure 1).

### Logistic regression models

Logistic regression models were developed with the dependent variable being the probability of dying up to seven days post discharge from hospital [24]. Three groups of models were developed using an array of independent variables: 1/a simple **background risk** model using only age and gender, 2/a group of **time-varying risk** models that estimated the risk associated with exposure to hospitalization (time of admission and a counter for number of days currently in hospital) and 3/a group of **disease specific models** which characterize specific risks associated with disease state (Charlson co-morbidity index), and subgroup analyses for 5 DRGs known to display significant day to day variation in risk of mortality [25]) as well as route of admission (Emergency Department (ED) or non-ED).

Within the time-varying risk model set, the differential impact of admission at nights and weekends was explored by developing three separate models that 1/treated all days as having equal risk, 2/distinguished the risk of weekdays from weekends, or 3/modeled both daily and weekly periodicities in the risk of death following admission. When sampling a sinusoidal function, to avoid aliasing or under sampling, the rate of sampling must exceed the Nyquist rate, and thus must be greater than double the frequency of the function being sampled [26]. As our mortality data has a daily periodicity of one cycle, our model would need to sample the distribution more than twice a day. We thus developed a model that segmented each day of the week into three sample periods: “daytime” (08:00 to 16:59 hours), “evening” (17:00 to 23:59), and “night” (0:00 to 07:59), creating 21 unique sample periods every week.

Finally, a **full logistic model** was assembled using these three separate models, which estimated the risk of exposure during hospitalization as the sum of background, time varying and disease specific factors:

$$\begin{array}{ll} \mathrm{Logit}\;\left(\mathrm{probability}\;\mathrm{of}\;\mathrm{death}\;\right)= & \beta_{1}\mathrm{Age}+\beta_{2}\mathrm{Gender}\\ & +\beta_{3}{\mathrm{comorbidity}}_{\mathrm{index}}\\ & +\beta_{4}\mathrm{Time}\;\mathrm{of}\;\mathrm{admission}\\ & +\beta_{5}{\mathrm{count}}_{\mathrm{MonDaytime}}\\ & +\ldots +\beta_{n}{\mathrm{count}}_{\mathrm{SunN}\mathrm{ight}} \end{array}$$

### Model training and testing

APDC data from 2001 to 2006 were used to train the logistic models (10,745,181 admissions), and 2007 data were set aside to prospectively test model performance (987,079 admissions). Models were developed using admission data and tested against their ability to correctly identify whether a patient was alive or dead at 7 days post discharge.

Model performance was assessed by the area under the receiver operating characteristic curve (AUC), also known as the C-statistic. Models with an AUC greater than 0.8 are considered to be good classifiers, with an AUC of 1 indicating perfect performance. In addition, we also estimated the ‘goodness’ of each model using the Akaike information criterion (AIC), and Bayesian information criterion (BIC) which are penalized model selection criteria that track model performance as the number of model parameters increase [27]. Both criteria help minimize over-fitting of models to data and amongst similarly performing models, the one with the smallest value is preferred.

AUC requires calculation of model sensitivity and specificity at different values of the ratio of patients who die to those who survive (the cut off value). We selected the optimal cut-off as that point in the ROC curve where the sum of sensitivity and specificity is maximal, and report sensitivity, specificity, positive predictive value (PPV), negative predictive value (NPV), and accuracy at this point. The study was approved by the NSW Population and Health Services Research Ethics Committee and the UNSW Human Research Ethics Committee.

## Results

The demographic distribution of patients in the training (July 2000 – December 2006) and test (2007) data sets is summarized in Table 1. Age and comorbidity patterns were similar in both groups. Mean length of stay dropped from 3.21 days in the seven training years to 2.75 in the final test year, and the death rate (the ratio of deaths during hospitalization and deaths within 7 days of discharge to all admissions) also dropped from 1.8% to 1.3%.

**Table 1:** *Demographic characteristics of patients in model training and testing data sets*

	**Training data (10,745,181 admissions)**		**Test data (987,079 admissions)**	
	**% of training data**	**Death rates**	**% of test data**	**Death rates**
Age distribution				
[0–5]	10.2%	0.3%	8.3%	0.2%
[6-15]	4.4%	0.1%	3.6%	0.1%
[16–35]	21.9%	0.1%	18.6%	0.1%
[36–55]	25.2%	0.5%	23.3%	0.4%
[56–65]	13.5%	1.4%	14.8%	1.0%
[66–75]	12.8%	2.8%	14.8%	1.7%
[76–85]	9.9%	6.1%	13.0%	3.5%
> = 85	2.2%	19.6%	3.7%	9.5%
Charlson score of comorbidity				
Zero to mild (0)	68.6%	0.2%	65.5%	0.2%
Mild [1,2]	20.0%	2.7%	20.2%	1.7%
Moderate [3,4]	6.8%	5.7%	8.5%	3.5%
Severe [> = 5]	4.5%	14.7%	5.8%	10.1%
Gender				
Female	54.7%	1.5%	53.6%	1.1%
Male	45.3%	2.1%	46.4%	1.6%
Overall death rates	1.8%		1.3%	
LOS				
Mean/Std. deviation	3.21/11.42		2.75/5.134	
Median	1.00		1.00	
Range	599		171	
Interquartile range	2		1	

### Model performance

When tested alone, individual model components for background risk, time-varying risk, and disease-specific risk had similar but modest power to identify risk of death up to 7 days post-discharge, with AUCs of 0.71, 0.79 and 0.79 respectively (Table 2). Amongst the three time- varying risk models, treating each day as if it has the same risk produced an AUC of 0.71, distinguishing weekdays from weekends 0.69, improving further to 0.79 with the fine-grained temporal model. This best temporal exposure model had a greater accuracy (74.4) compared to the background risk (66.2) and illness severity (66.6) models alone.

**Table 2:** Model Performance in predicting death up to 7 days post-discharge, as measured by AUC, AIC and BIC

**Model type**	**Variables used**	**Number of variables**	**AUC**	**AIC**	**BIC**	**Optimal cut-off point (ratio of deaths to survival)**	**Sensitivity**	**Specificity**	**PPV**	**NPV**	**Accuracy**
**Model 1:** Background risk	Age, Gender	2	.711 (.708 -.715)	1.0710e + 006	1.0710e + 006	0.196	71.29	66.14	2.78	99.41	66.21
**Model 2:** Time exposure risk	2(a): Length of stay (LOS)	1	.710 (.705 - .714)	1.3155e + 006	1.3155e + 006	0.168	69.07	73.69	3.45	99.43	73.62
	2(b): LOS (Weekday, weekend), admission time	3	.692 (.687 - .697)	1.1960e + 006	1.1961e + 006	0.145	71.97	70.21	3.18	99.5	70.23
	2(c): LOS (Morning, evening, or night for each of seven days), admission time	22	.786 (.782 - .79)	1.3464e + 006	1.3467e + 006	0.228	64.62	74.57	3.34	99.4	74.44
**Model 3:** Disease model	Charlson comorbidity index	1	.786 (.783 - .79)	1.0826e + 006	1.0826e + 006	0.1	91.54	66.27	3.56	99.8	66.60
**Model 4:** Background plus time exposure risk	4(a): 1 + 2(a)	3	.73 (.726 - .739)	0.9408e + 006	0.94097e + 006	0.196	76.14	61.25	2.6	99.5	61.5
	4(b): 1 + 2(b)	5	.743 (.739 - .747)	1.1519e + 006	1.1520e + 006	0.227	79.95	68.72	3.36	99.6	68.87
	4(c): 1 + 2(c)	24	.883 (.88 - .886)	1.4713e + 006	1.4716e + 006	0.32	82.41	77.41	4.73	99.7	77.47
**Model 5:** Full model	5(a): 1 + 2(a) + 3	4	.891 (.888 - .894)	1.5143e + 006	1.5143e + 006	0.197	87.09	78.32	5.18	99.8	78.43
	5(b): 1 + 2(b) + 3	6	.893 (.89 - .896)	1.5118e + 006	1.5119e + 006	0.224	86.86	78.27	5.16	99.8	78.38
	5(c): 1 + 2(c) + 3	25	.923 (.921 - .926)	1.6769e + 006	1.6772e + 006	0.271	88.18	81.61	6.13	99.8	81.71
**Model 6:** DRG-specific models 5(c) trained and tested only single DRG subpopulation	R61: Lymphoma and Non-Acute Leukemia	25	.90 (.878 - .922)	4.7669e + 003	4.9223e + 003	0.149	81.45	82.63	34.67	97.5	82.51
	E02: Other Respiratory System OR Procedures	25	.940 (.911 - .969)	2.6019e + 003	2.7281e + 003	0.398	80.0	95.25	21.05	99.6	94.99
	F70: Major Arrhythmia and Cardiac Arrest	25	.762 (.736 - .802)	982.0188	1.1044e + 003	0.412	75.86	65.79	68.75	73.3	70.81
	E64: Pulmonary Oedema and Respiratory Failure	25	.85 (.807 - .894)	466.95	476.32	0.616	71.34	88.41	87.5	73.05	79.32
	J62: Malignant Breast Disorders	25	.903 (.874 - .933)	704.03	713.95	0.438	77.69	86.49	74.26	88.54	83.55
**Model 7:** Emergency Department admission 5(c) trained and tested only on ED or non ED subpopulations	ED admission	25	.909 (.905 - .912)	3.0017e + 005	3.0044e + 005	0.16	87.80	77.32	11.91	99.45	77.67
	Non-ED admissions	25	.925 (.921 - .928)	1.4626e + 006	1.4629e + 006	0.218	87.70	84.38	4.47	99.9	84.41

An optimal cut-off value is selected as the point in the receiver operating characteristic curve where the sum of sensitivity and specificity is maximum. Sensitivity, specificity, model accuracy, positive predictive value (PPV) and negative predictive value (NPV) are reported at the optimal ratio of patient deaths to survival.

When the individual models were combined to create the full model, AUC rose to 0.92 for the whole population. When the model was trained and tested only on patients admitted via the Emergency Department (ED) the AUC was 0.91; training and testing on non-ED admissions achieved an AUC of 0.92. Performance dropped to 0.62 when the population-trained model was tested only on patients with a primary diagnosis of a major arrhythmia or cardiac arrest, 0.75 for Lymphoma and Non-Acute Leukaemia and 0.87 for laryngoscopic, mediastinal and other chest procedures. When the full model was trained using only the subgroup of patients from these DRGs, performance improved, but variably. Patients with major arrhythmia or cardiac arrest had minimal improvement (AUC 0.76). Lymphoma and Non-Acute Leukaemia patients (AUC 0.90), and malignant breast disorders (AUC 0.90) improved substantially, and patients with laryngoscopic, mediastinal and other chest procedures surpassed the general population benchmark (AUC 0.94).

**Figure 2:**
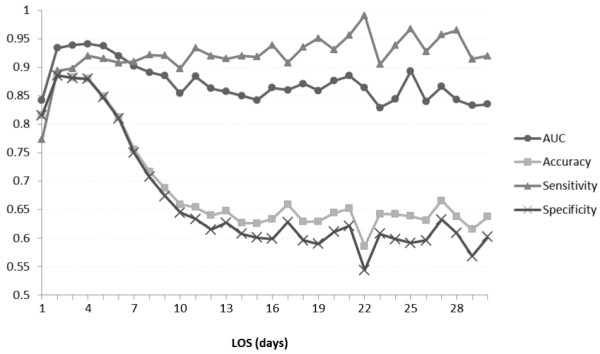
Performance of the ‘full model’ as a function of length of stay (LOS).

The combined or full model’s performance varied with length of stay (Figure 2). The model performed best with patients of hospital stays of seven days or less, covering some 93% of admissions, and peaking at days 3 to 5 with an AUC of 0.94. After seven days, model sensitivity remained surprisingly steady, but specificity dropped, indicating that model performance was deteriorating because of an increased number of false positives. Performance also became increasingly erratic, possibly reflecting smaller patient numbers both in training and tests sets, as well as increasing influence of unmodelled disease and service specific factors.

AIC and BIC measures closely tracked each other for all models, and showed an increase with increasing model complexity, as expected. Most models had AIC values in the range 1.071-1.68e + 006 except for the more specialised models based upon single DRG (e.g. R61: Lymphoma and Non-Acute Leukemia, AIC = 4.7669e + 003).

## Discussion

Modeling the time accumulated risk associated with hospital stay has allowed the development of a class of predictive models that, when combined with simple disease data appears to substantially outperform many disease specific models alone. This is despite the disease specific information used in our model being represented by relatively simple linear combination of administrative information including the Charlson index and DRG categories. One would anticipate even better performance if an exposure model was combined with a clinically-based disease prognostic model, relying for example on patient record or disease registry data, as well as better modeling the relationship of variables to mortality (e.g. age and death are better represented as a log linear rather than linear relationship [28]).

**Figure 3:**
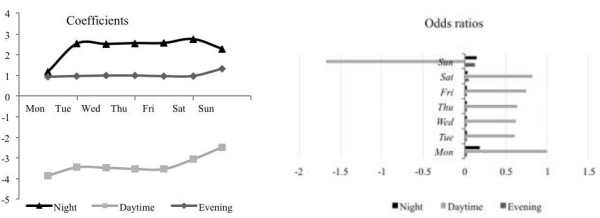
Coefficients for the contribution of different time periods to the full model (left) and corresponding odds ratios for risk of death following admission at a different time of the week, compared to Monday day as a reference (right).

A striking feature of our data set is the strong daily rhythm to risk of death, which echoes the well-known weekly variation of risk of death, which peaks with weekend admission (Figure 1). Our data shows the risk of mortality peaks in the early evening and is at its lowest in the morning (Figure 3). Daycare did not appear to increase risk of death but this attenuated at weekends – the weekend effect. Compared to admission on Monday day, the odds ratios for risk of death were worse for evenings and nights, and Sunday daytime had a 1.7 times greater risk than Monday daytime.

It is hard to impute causation to such observational data and we can only speculate why the diurnal rhythm exists. There are two main causal readings to be explored. Firstly, it may be that such risks of death are a feature of the health service, where patients experience increased risk because of reduced availability or quality of clinical services. The push for health services to provide uniform “24/7” care is a response to this concern. The second causal reading is equally plausible, and that is that some patient groups are associated with greater risk of death at different times. This may be due to a selection bias, where sicker patients present to hospital at given times, or in some instances may have a biological cause. Our recent analysis of the weekend effect identified that both causal readings are plausible, and that different patient groups demonstrate either care or illness risk patterns [25].

We show that performance of models using patient subgroups based upon DRGs is also variable. For example, performance was not strong for patients with major arrhythmia or cardiac arrest. As 8,254 admissions with major arrhythmia or cardiac arrest were present in the training data set, with an average LOS of 2.69, it seems unlikely that sample size or LOS were factors influencing performance. In contrast model performance was strong for DRGs associated with leukemia or lymphoma. In our recent analysis of weekend effects, increased weekend deaths for acute cardiovascular events appear to be related to service quality (e.g. lack of availability of imaging or stenting services out of hours). For oncological patients however, the cause seems to be that a sicker cohort of patients is presenting at the weekend [25]. This suggests that the models developed here may be better at modeling disease rather than service specific factors in its exposure risk profile, and warrants further investigation. Where there are time varying risks associated with a health service for a particular patient group, specific modeling of that independent risk may be needed.

Modeling the route of admission (Emergency vs. non-Emergency Department) did not appear to confer any advantage despite it being show by others to be a major predictor of mortality [9]. One possible explanation is that information about route of admission is already captured implicitly in the exposure model. For example, patients admitted out of hours may be most likely to presenting via emergency, and so the out of hours risk is already modeling route of admission as a hidden covariate. Further work is needed to understand the nexus between time-varying risk and route of admission.

Adding new variables to the basic model helped improve model accuracy at the expense of increasing AIC and BIC values (Table 2). However AIC/BIC value increments were modest and the improvement in model performance substantial. Further, interpreting AIC and BIC values is hard with large data sets, and they typically are more valuable when models are developed on small data sets. With large data sets, AIC and BIC may end up preferring over-fitted models [24].

Most current clinical predictive tools provide snapshot predictions of risk, independent of time. Yet clearly risks should vary with time. In this study predictive performance was best in the first week of admission, and performance early in the first week was exceptionally strong. One would expect as admission time lengthens that many additional factors come into play to modify risk, and therefore long run prediction becomes increasingly difficult – a situation well known in forecasting. It is also the case that patient mix changes with time, and those patients with long stays represent a different cohort with different risk profile, and may need to be modeled separately. Sample sizes available for such model development also diminish as length of stay increases, making it harder to develop generalizable models.

**Figure 4:**
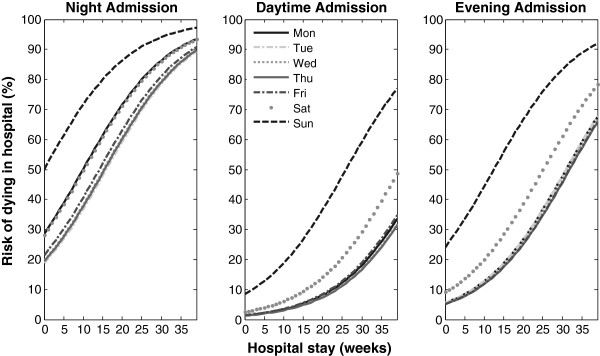
Example prediction of the time varying risk of death over time for a simulated patient, showing variation in risk curves depending on day and time of initial admission.

Models such as those presented here could be used to forecast risks such as death or specific clinical events, and to update those forecasts as additional information accumulates. Using the full model developed here, it is clear that the risk profile for patients varies with both time and day of admission (Figure 4). Given that predictive accuracy changes with LOS, it would also be appropriate to provide clinicians with estimates of the accuracy of each prediction and emphasize for example that confidence is stronger in the short rather than long run. The next stage in developing the models presented here would be to evaluate their capacity to forecast future risk of death for patients given their current exposure to hospitalization.

One challenge for creators of clinical prediction models is that they often fail to generalize to settings beyond those from which they are created, because they model a point in time, local disease patterns or service provision patterns [1]. The approach developed here is likely to be highly generalizable as it relies on no location specific information, except for the weightings associated with different model variables. These weightings could be calibrated using the best available data for a given location. Further, the evaluation here was on patients from a large geography encompassing urban, rural and remote settings, and hospitals ranged from public to private, and small to large teaching and specialist referral centers. Addition of location specific variables and disease specific elements are likely to further enhance the general model reported here.

## Conclusions

Risks of death associated with hospitalization vary not just with the day of the week but also time of the day, and can be used to make predictions about the cumulative risk of death associated with an individual’s hospitalization. Enhancing disease specific prognostic models with estimates of the cumulative risk of exposure to hospitalization appears to greatly enhance predictive performance. Risk exposure models should find utility both in enhancing standard prognostic models as well as estimating the risk of continuation of hospitalization.

## Abbreviations

AIC: Akaike information criterion; APDC: Admitted patient data collection; AUC: Area under receiver operating characteristic curve; BIC: Bayesian information criterion; DRG: Diagnosis related group; ED: Emergency department; ICD-10-AM: International classification of diseases 10th revision Australian modification; LOS: Length of stay; NPV: Negative predictive value; NSW: New South Wales; PPV: Positive predictive value.

## Competing interests

The authors declare that they have no competing interests.

## Authors’ contributions

YW has had full access to all the data in the study and takes responsibility for the integrity of the data and the accuracy of the data analysis. *Study concept and design*: EC and YW. *Acquisition of data*: BG and OC. *Analysis and interpretation of data*: EC, YW, FM, BG, OC and WR. *Drafting of the manuscript*: EC and YW. *Critical revision of the manuscript for important intellectual content*: FM, BG, OC and WR. *Statistical analysis*: YW. All authors read and approved the final manuscript.

## Pre-publication history

The pre-publication history for this paper can be accessed here:

http://www.biomedcentral.com/1472-6963/14/226/prepub
